# The impacts of ready-to-eat-cereals and cereal fibers on gut health, body weight, and cardiometabolic health

**DOI:** 10.3389/fnut.2026.1717345

**Published:** 2026-04-17

**Authors:** Kevin B. Comerford

**Affiliations:** OMNI Nutrition Science, Woodland, CA, United States

**Keywords:** blood glucose, cardiovascular disease, gut microbiome, insoluble fiber, obesity, prebiotic fiber, type 2 diabetes, wheat bran

## Abstract

Ready-to-eat breakfast cereals are a major source of dietary fiber, and their intake is associated with better diet quality and reduced incidence of chronic disease. However, dietary fiber intake remains significantly lower than recommended levels, particularly in North America. This fiber gap is one of the most important issues facing public health nutrition and deserves continued attention. This extensive analysis summarizes the body of research from the last decade on whole grain/high-fiber breakfast cereals, cereal fibers, and/or selected fiber sources commonly found in, or added to, breakfast cereals (e.g., wheat bran, psyllium). The primary health outcomes of interest for this review are digestive function, gut microbial effects, satiety signaling, body weight management, cardiovascular disease and blood glucose control. The evidence indicates that the fiber amount, fiber type, processing techniques, and numerous associated nutrients and phytochemicals in ready-to-eat breakfast cereals are all critical factors impacting health outcomes. Therefore, in addition to dietary guidance on total daily intake levels, guidance targeting specific health outcomes should also emphasize the unique mechanisms of action (e.g., gel-forming, digestion slowing, fecal-bulking, laxative, toxin binding, prebiotic) for the predominant types of fibers in ready-to-eat cereals and other fiber-rich foods. In particular, a growing body of research indicates that wheat bran, the predominant source of fiber in the U.S. and Canada, contains a novel array of fibers and phytonutrients that support bowel function and influence gut microbiota composition, and may help lower the risk for cardiometabolic disease. Notably, the research shows that individuals with low-cereal fiber consumption are most likely to benefit from an increase in their daily intake. While there is still much to discover regarding the mechanistic effects of different types of cereal fibers, continued encouragement to increase daily consumption of wheat fiber-rich foods, including ready-to-eat cereals, could help to close the fiber gap and reduce the incidence of multiple diet-related chronic diseases.

## 1 Introduction

Although dietary fiber is abundant in a variety of commonly consumed foods, total intake remains significantly lower than recommended levels throughout most of the world (1–3). In the U.S., the National Academy of Medicine established an adequate intake of fiber for women of 25 g/day and for men of 38 g/day based on a 2,000-calorie diet (4). However, many Americans only consume a little more than half of the recommended amount per day (5, 6). This fiber gap is one of the most important issues facing public health nutrition. Ready-to-eat cereals (RTECs) are a primary source of dietary fiber in North American and European diets, contributing ~6% of total fiber in the U.S. (7) and ≥20% of total fiber intake in certain populations in Canada and Europe (8, 9). Observational studies show that regular RTEC consumption has consistently been linked with higher fiber intake, higher micronutrient intakes, and better diet quality when compared to populations that regularly skip breakfast or consume non-RTEC breakfasts (10–14). For more than half a century, the beneficial effects of cereal fiber consumption on human health have been reported (15, 16). And for the last several decades, researchers have continued to provide evidence for the benefits of higher fiber intake, especially for cereal fibers, on an array of health outcomes, with the greatest effects reported among low-fiber consumers and consumers of Western-style diets (17–19). The most well-known benefits of adequate fiber intake involve the modulation of gut motility and bowel health, with other potential lesser-known benefits on satiety/food intake, body weight regulation, and cardiometabolic health (20). Despite the known benefits of dietary fiber, many of the mechanisms remain elusive. Among others, significant knowledge gaps still exist regarding different food sources of fiber, different functional properties of fibers, different effects of food processing on fibers, and the importance of the food matrices in which fibers are delivered.

Dietary fiber can be obtained from different sources, including cereals, fruits, vegetables, pulses, nuts, and seeds. The types and amounts of fiber in these foods can vary considerably, with most foods containing multiple types of fiber that each possess unique functional properties (Table 1). Solubility, viscosity, and fermentability are three of the most important factors dictating the functional properties of dietary fibers. Soluble fibers, which make up the dominant sources of fibers in oats, fruit, and psyllium, tend to have the greatest prebiotic effects, meaning they can be readily fermented by gut microbiota, impacting gut microbial composition and the microbial production of vitamins and metabolites such as short-chain fatty acids (SCFAs) (19). Soluble fibers can be further divided into viscous or non-viscous fibers. Viscous soluble fibers from psyllium and oats (e.g., beta-glucan) are able to absorb water and form gels that extend the intestinal transit time of foods and tend to slow down the rate of fermentation relative to non-viscous soluble fibers. Insoluble fibers, such as those primarily found in rice, corn, nuts, seeds, and vegetables tend to have more localized effects in the gastrointestinal tract such as by increasing fecal-bulking and fecal transit time, which can help promote bowel movement regularity and prevent constipation (19, 21). Notably, wheat fibers are unique among dietary fiber sources in that they are largely comprised of insoluble and non-viscous fibers that can both add fecal bulk (without forming gels) while also being fermentable by gut bacteria (22). Most cereal grains contain unique mixes of soluble and insoluble fibers, viscous and non-viscous fibers, and fermentable and non-fermentable fibers; and the particular combinations of fibers as well as how they have been processed likely contribute to their differing impacts on health (22). Overall, RTECs may contain a heterogenous mix of fibers and other ingredients (including added sweeteners, oils, preservatives, and/or micronutrient fortifications) that can impact their functional properties, making it difficult to extrapolate research findings from one RTEC to the next.

**Table 1:** Food sources and functional properties of common fibers in ready-to-eat cereals.

Food source	Primary fiber type(s)	General functional properties
Wheat	Wheat Bran: Primarily comprised of AXOS, followed by cellulose, lignin and beta-glucan.	Mostly insoluble, with some soluble beta-glucan. Mostly non-viscous, with some viscous beta-glucan. Fermentable and non-fermentable components. High-water binding capacity. Low gel-forming.
Oat	Oat Bran: Mostly comprised of beta-glucan, followed by cellulose, hemicellose and lignin.	Mostly soluble. Mostly viscous. Mostly fermentable. High water and fat binding capacity. High Gel-forming.
Rice	Rice bran: Mostly comprised of cellulose, hemicellose and lignin.	Mostly insoluble, with some soluble components. Mostly non-viscous. Mostly non-fermentable. High-water and moderate fat binding capacity. Moderate gel-forming.
Corn	Corn bran: Mostly comprised of cellulose, hemicellulose and lignin.	Mostly insoluble, with some soluble components. Mostly non-viscous. Mostly fermentable. Moderate water binding capacity. Low gel-forming.
*Plantago Ovata* Seed	Psyllium Husk	Mostly soluble. Mostly viscous. Mostly non-fermentable. High-water and fat binding capacity. High Gel-forming.

Approximately a decade ago, two systematic reviews on RTECs and general health were published (23, 24). The first review focused on oats/oatmeal and wheat-based cereals and concluded that breakfast cereal consumption may be associated with improved bowel function, lower risk for obesity, lower cholesterol levels, and lower risk for type 2 diabetes (T2D), but these effects largely depended on the type of cereal consumed, with wheat- and oat-based cereals each showing unique effects on health outcomes (23). The second review primarily focused on wheat-based RTECs and concluded that higher RTEC consumption was associated with reductions in hypertension and T2D risk, while the effects of RTECs on body weight and intestinal health needed further evaluation (24). The purpose of the current review is to update and expand on the evidence from these prior publications on wheat- and oat-based RTECs, by integrating information from dozens of additional systematic reviews focused on whole grain/high-fiber breakfast cereals, cereal fiber, and/or selected fiber sources commonly found in, or added to, breakfast cereals (e.g., wheat bran, psyllium). The primary health outcomes of interest for this review are digestive function, gut microbial effects, satiety signaling, body weight control, cardiovascular disease (CVD), and blood glucose control and T2D risk.

## 2 Methods

### 2.1 Search strategy

The initial literature search was focused on systematic reviews and/or meta-analyses published between 2014 and 2024 in PubMed, Web of Science, Embase, and Google Scholar. The searches were split into four parts for the independent variables: (1) ready-to-eat cereals, (2) cereal fibers, (3) wheat fiber and wheat bran, and (4) isolated fibers used in breakfast cereals, i.e., AXOS and psyllium fiber. Systematic reviews and/or meta-analyses on total dietary fiber intake that showed up in the searches were considered for inclusion if they provided specific information on cereal fiber or wheat fiber intake. In combination with the independent variables, searches were conducted for systematic reviews and/or meta-analyses for each of the six dependent variable of interest and their associated outcome measures, which included (1) “Bowel Function,” – “digestion,” “bowel movement,” “stool frequency,” “constipation,” “diarrhea,” “stool or fecal bulk,” “stool or fecal weight,” “stool water-binding capacity,” “intestinal motility,” “intestinal transit time,”; (2) “Gut Microbial Effects” – “gut microbiota or microbiome,” “gut microbial diversity,” “gut microbial abundance,” “gut microbial metabolites,” “fermentation metabolites,” “colonic fermentation,” “prebiotic effects,” “short-chain fatty acids”; (3) “Satiety Signals and Food/Energy Intake” – “satiety,” “appetite,” “hunger,” “food intake,” “energy intake,” “GLP-1”; (4) “Body Weight Regulation,” -“weight management,” “weight reduction,” “overweight,” “obese/obesity,” “BMI,” “body composition,” “adiposity,” “fat mass,” “waist circumference,”; (5) “Cardiovascular Disease,” – “blood lipids,” “triglycerides,” “hyperlipidemia,” “lipoprotein,” “cholesterol,” “blood pressure,” “hypertension,” “coronary heart disease,” “ischemic heart disease,” “myocardial infarction,” “stroke,” “cardiometabolic”; and (6) “Blood Glucose Management and Type 2 Diabetes” – “blood glucose or sugar”, “glycemic/glycemia,” “hyperglycemia,” “insulin,” “insulinemia” “hyperinsulinemia,” “HOMA-IR”, “hemoglobin A1C,” “pre-diabetes,” “type 2 diabetes.” If a systematic review and/or meta-analysis covered more than one topic area (e.g., obesity and cardiovascular disease) it was reviewed for both topic areas. Additional searches were conducted for studies published after the most recently published systematic reviews and/or meta-analyses for each section topic. For example, if the most recent systematic review for “Bowel Function” was published in 2016, then searches were conducted for individual studies published between 2017 and 2024 to provide a more current update on the literature. Reference lists for all articles were checked for additional relevant publications using the snowball method.

## 3 Gut health

In recent years, gut health has emerged as a key factor linking diet and overall health. Gut health research now includes topics covering bowel function and waste removal, intestinal barrier integrity, intestinal immunity, and the remodeling of the gut microbiome (25). While all of these aspects are interdependent and important for overall health, there are two outcomes that are most frequently addressed in the scientific literature in regard to RTECs, cereal grains, and/or fiber intake: these are bowel function and gut microbiome composition. Although the impacts of cereal fiber consumption on bowel function and gut microbiome composition are inextricably linked, the research literature tends to address these topics separately. The research to date on cereal fiber intake and bowel function primarily focuses on insoluble/non-fermentable fibers that impact bowel movement regularity, stool weight, and total transit time (Table 2); whereas the research on cereal fiber intake and gut microbial effects generally focuses on the intake of soluble/fermentable fibers (i.e., prebiotic fibers) that can alter gut microbial composition and the microbial metabolites that are produced (Table 3). Since these topics largely depend on two different bodies of evidence, they are addressed as separate sections below.

**Table 2:** Bowel function – systematic reviews and/or meta-analyses.

Author, year	Articles included	Population	Food/fiber type	Measures	Findings
Williams 2014 (23)	5 intervention studies	Healthy adults	Ready-to-eat cereals	Stool frequency, stool bulk, stool transit time	Ready-to-eat cereals associated with increased stool frequency and decreased laxative use.
De Vries et al. 2015 (38)	65 intervention studies	Healthy adults	Intact cereal fiber; primarily wheat fiber	Stool weight, percentage stool water, stool frequency and consistency, stool transit time	Intact cereal fiber associated with increased stool weight and stool frequency, and decreased total intestinal transit time.
De Vries et al. 2016 (5)	136 intervention studies	Healthy adults	Cereal, fruit, and vegetable fibers	Fecal weight, stool transit time	Cereal fiber associated with increased fecal weight and decreased intestinal transit time.

**Table 3:** Gut microbial effects – systematic reviews and/or meta-analyses.

Author, year	Articles included	Population	Food/fiber type	Measures	Findings
Jefferson, et al. 2019 (22)	40 intervention studies	Healthy adults	Intact cereal fibers, including wheat bran	Gut microbiota composition	Intact cereal fiber associated with increased microbiota diversity and/or abundance.
Williams et al. 2021 (62)	46 intervention studies	Healthy adults	Cereal fiber	Gut microbiota composition, short-chain fatty acids	Cereal fiber associated with changes in microbiome composition. Heterogenous effects on short-chain fatty acids.
Vinelli et al. 2022 (63)	44 intervention studies	Healthy adults	Dietary fiber; including wheat fiber, wheat bran, and AXOS	Gut microbiota composition, short-chain fatty acids	Dietary fiber associated with changes in microbiome composition. Heterogenous effects on short-chain fatty acids.

### 3.1 Bowel function

Constipation is the most common chronic gastrointestinal disorder in adults (26). Approximately 10 to 20% of U.S. adults experience chronic constipation (27), and each year in the U.S., constipation accounts for around three million visits to medical centers (28). Low fiber consumption is one of the primary factors associated with chronic constipation, and higher fiber intake is consistently recommended by nutrition and health professionals as the first line of management for improving bowel movement regularity (29). In addition to increasing the total amount of fiber consumed, research also indicates that special attention should be paid to the specific type(s) of fiber being recommended to reduce constipation, since different sources of fiber can have very different effects on bowel regularity.

Wheat bran is the most studied source of cereal fiber in regard to digestive health, and stands out from other cereal fibers due to its unique combination of largely insoluble, yet fermentable fibers and prebiotic phenolic compounds (e.g., ferulic acid) (22, 30). Both of these properties together may help to increase intestinal motility which can reduce stool transit time (5). However, the way that wheat bran has been processed may dramatically impact its effects on stool water content and hardness, as well as its effects on the gut microbiome (31–33). For viscous soluble fibers, such as those from oats and psyllium, their unique functional properties such as their fermentability and water-holding capacity may also contribute to bowel function regularity, and may be especially useful in slowing down digestive processes associated with diarrhea (34).

In 1993, Cummings reported on data from nearly 100 intervention studies which investigated the role of dietary fiber on fecal weight (35). These studies spanned 60 years of research on different sources of fiber with and included more than 40 studies focused specifically on wheat fiber and/or wheat bran. The evidence from this comprehensive review indicated that the consumption of wheat fiber resulted in higher average increases in fecal weight than other fiber sources. For example, for every gram of wheat fiber consumed there was an average increase of 5.4 g in fecal weight. For fruits and vegetables, the average increase was 4.7 g, and for cellulose, oats, and corn it was around 3.5 g for every gram of fiber consumed. In 2010, the European Food Safety Authority (EFSA) was of the opinion that the evidence for wheat bran fiber was strong enough to substantiate two health claims for the general population, one for the intake of high-wheat fiber foods and fecal bulking and the other for consuming ≥10 g/day of wheat bran for reducing intestinal transit time (36). A later comprehensive review by O’Sullivan in 2012, further reported that among low-fiber consumers, bowel health benefits in the form of reductions in self-reported levels of constipation and in the use of laxatives were seen within a few days of consuming higher levels of wheat bran (37).

A 2014 systematic review by Williams which focused on the role of RTEC consumption on general health, reported strong evidence from 5 RCTs that regular consumption of high-fiber, wheat-based breakfast cereals improved bowel function and prevents constipation (23). All the RCTs in the 2014 Williams publication reported improvements in stool frequency, bulk, and/or transit time from high-fiber cereal consumption. Stool frequency increased by at least 25% on average in the RCTs, and multiple studies reported that participants in the high-fiber RTEC interventions needed fewer laxatives to maintain bowel regularity. Although this systematic review only included a handful of RCTs focused on breakfast cereal consumption, the outcomes from these studies consistently showed beneficial associations between high-fiber wheat-based RTECs and bowel function.

In accordance with these findings, a 2015 systematic review of 65 intervention trials on healthy adults found that for each extra gram per day of intact wheat fiber consumed (not specifically from breakfast cereals), there were significant increases in stool weight and stool frequency, and significant reductions in total intestinal transit time (38). In this review, stool weight was shown to be increased by nearly 4 g for every 1 g of intact wheat fiber consumed. Notably, intestinal transit time was shown to be reduced by approximately 45 min per gram of intact wheat fiber consumed, but only among individuals with an initial total transit time of 48 h or more. Reductions in intestinal transit time were not seen for individuals with a total transit time of less than 48 h. The normal physiological range for total transit time for healthy adults varies between 14 to 60 + hours (5, 39, 40), indicating that individuals who are at the higher end of this “normal” range may see improvements in intestinal function from as little as a 1 gram increase in fiber consumption per meal (38).

Since 2016, few studies have focused on the effects of RTECs or cereal fibers on bowel function. The majority of studies have simply focused either on total dietary fiber intake (41–43), whole grain intake (44–46), and/or on the wheat bran extract known as arabinoxylan-oligosaccharides (AXOS) (47). These publications, which include a mix of both observational and clinical studies, consistently indicate that higher intake of various types of fiber can help improve stool consistency, reduce constipation and reduce chronic diarrhea. These studies also reveal multiple consumer traits and characteristics moderating the relationships between dietary fiber intake and bowel function. For example, an analysis of 2005–2010 NHANES data showed that higher fiber intake only improved stool consistency related constipation among physically active subjects, but not among low-activity subjects (41). Another analysis of the 2005–2010 NHANES data showed that higher dietary fiber intake was associated with reduced incidences of chronic diarrhea, but only in women who consumed greater than 25 g fiber/day (42). Similarly, results from an RCT on healthy adults indicated that higher AXOS supplementation may only improve stool transit time in individuals who had slow intestinal transit time to begin with Muller et al. (47). The body of research also revealed contextual variables having to do with the processing and/or delivery of fiber in the diet, with intrinsic wheat fiber delivered within a solid-form food matrix having more potent effects on fecal bulking than extrinsic wheat fiber delivered within the context of a liquid-form food matrix such as a fiber-enriched beverage (48).

One of the only studies published since 2016 which focused on high-fiber RTEC consumption and gastrointestinal (GI) symptoms was an RCT that focused on a very specific population of individuals who were fasting for large parts of the day in accordance with the Islamic religious holiday Ramadan (49). This type of fasting was studied since it has been associated with increased rates of constipation and other negative effects on GI hormones. The study showed that individuals who consumed 90 g of high-fiber cereal (11 g fiber/serving) each day at their pre-dawn/pre-fasting meal for 20 consecutive days had improved bowel movements compared to individuals who maintained their regular low-fiber breakfast meal for those 20 days.

Taken together, these findings indicate that different types of dietary fibers may have different health benefits according to the characteristics of the fiber such as whether the fiber is soluble or insoluble, viscous or non-viscous, fermentable or non-fermentable, part of the bran or whole grain, how finely the bran has been ground, the food matrix the fiber is consumed in, as well as the characteristics of the consumer (e.g., activity levels, sex, average GI transit time). At present, the best available observational and clinical evidence indicates a variety of fiber types, including many different wheat-based fiber sources (e.g., whole grain wheat, wheat bran, AXOS), oats and psyllium are associated with improved bowel function. These benefits appear to be greatest among low fiber consumers, which include most Americans (50).

### 3.2 Gut microbial effects

The human gut microbiota and its array of associated metabolites have been increasingly recognized as critical factors impacting human health and disease. Potential benefits associated with improved gut microbial abundance and/or diversity include improved digestive health, improved immune function, improved vitamin synthesis and mineral bioavailability, and reductions in obesity, CVD, T2D, and certain types of cancer (22). Whole grains are an important dietary source of fermentable compounds that can be used by the gut microbiota. These compounds are generally concentrated in the bran layer, and may include multiple types of fibers, phenolic compounds, protein fractions, lignin, waxes, saponins, and phytates (51). Some research suggests that bran consumption may provide most of the same health benefits as whole grain consumption due to bran’s high concentration of prebiotic compounds, essential fatty acids, micronutrients, and phytochemicals (e.g., flavonoids, carotenoids, alkylresorcinols) (22, 32, 52–54). Wheat bran in particular is a concentrated source of AXOS, which are a heterogenous group of fermentable oligosaccharides that have been studied extensively for their potential gut protective effects (22, 53). The AXOS in wheat bran have a close physio-chemical association with phenolic compounds such as ferulic acid (22). These phenolic acids act as antioxidants and have anti-inflammatory properties that may complement or potentially synergize with the effects of certain fibers to impact gut health (55).

Until the last decade or so, wheat fiber consumption has been thought to have minimal effects on the gut microbiota due to its predominance of insoluble fibers (22), however, a growing body of evidence now indicates that higher intake of wheat-fiber-rich foods, such as RTECs which have been enriched with wheat bran, influence the gut microbiota due to their variety of fermentable constituents (30). For example, in 2014, a systematic review of mostly observational studies that focused on RTEC consumption and general health found limited research available at the time on the effects of breakfast cereal on the gut microbiome (23). Likewise, a 2016 systematic review of both observational and intervention studies on RTECs and health outcomes (24) drew similar conclusions from the evidence, concluding that the effects of RTECs on intestinal health required further evaluation.

It wasn’t until 2019, that wheat fiber/wheat bran was broadly shown to have important effects on gut microbial composition, when a systematic review by Jefferson and Adolphus summarized the evidence from 40 intervention trials investigating the effects of higher intake of intact cereal fibers, wheat bran, or AXOS on gut microbiota composition in healthy adults (22). Of the 40 studies included in the systematic review by Jefferson and Adolphus, 27 focused on wheat fiber, with 17 involving higher intake of wheat fiber, wheat bran and/or AXOS containing foods such as RTECs. Nearly all of the studies included in the systematic review were short-term, ranging between 1 day and 3 months, and only one study lasted for more than a year. Furthermore, most studies included populations with large inter-individual variability in baseline gut microbiome composition and large ranges of in fiber dosing, making it difficult to quantify the prebiotic effects of different RTECs, intact cereal fibers, and isolated fibers on the gut microbiome. Wheat fiber dosages ranged from 6 to 21 g/day, wheat bran dosages ranged from 13 to 28 g/day, and AXOS dosages ranged from 2 to 19 g/day. Despite the major differences in study designs included in the systematic review, nearly all studies reported that the intake of intact cereal fiber from RTECs and other foods resulted in prebiotic effects that increased microbial abundance, microbial diversity, and/or colonic fermentation metabolites relative to the lower-fiber consumers (22). These findings are all the more notable given that prebiotic effects were seen with wheat fiber dosages as low as 6 g/day, with significant increases in beneficial gut bacteria reported for *Bifidobacteria* and *Lactobacillus* species among others. Overall, this systematic review consistently showed prebiotic effects of wheat fiber and AXOS consumption, even when doses in the lower range of the intervention were provided at a single meal (e.g., ≥6 g of wheat fiber, or ≥2.2 g of AXOS).

Several studies that were summarized in the 2019 the Jefferson and Adolphus systematic review (22) also showed that AXOS intake has been associated with dose-dependent effects on *Bifidobacteria* growth, with stepwise increases in growth shown when moving from the consumption of 2.2 g to 5 g/day, and then again to ≥8 g/day of AXOS (56–59). At higher levels of AXOS intake (i.e., ≥8 g/day), significant increases in other fermentation metabolites such as circulating SCFAs have also been reported (60). Higher levels of SCFAs are involved in the regulation of immunity, inflammation, and lipid metabolism, and are likely one of the key mediators linking improvements in gut health to overall health (61). Therefore, there may be multiple systemic health benefits that are associated with the prebiotic effects of AXOS-containing foods. Overall, the wheat fiber induced stimulation of *Bifidobacterial* growth and SCFA production in the human intestinal tract appears to be most pronounced in study participants who had the lowest baseline fiber intake and lowest commensal gut bacterial abundances to begin with, suggesting that low-fiber consuming populations will likely experience the greatest potential health benefits from increasing their intake of intact cereal fibers, wheat bran, and/or AXOS (22). However, longer term studies, and a more detailed understanding of gut microbial composition changes are required to fully understand the role that RTECs and cereal fibers play in gut microbial health.

Since the 2019 systematic review by Adolphus and Jefferson (22), a few additional studies have directly addressed the topic of cereal fiber intake and the gut microbial effects. In 2021, a systematic review of grain-based fibers and the gut microbiome found that while cereal fiber modulated the microbiome, this had heterogenous impacts on SCFA production (62). In 2022, a systematic review of 42 RCTs, which included studies on wheat fiber, wheat bran, and AXOS, reported a trend towards increases in *Bifidobacterial* proportions and SCFAs with as little as 2.2 g/day of AXOS (63). In 2022, a four-week long RCT of 69 healthy men demonstrated that when compared to placebo, daily consumption (55 g) of a breakfast cereal rich in extruded wheat bran led to serum increases in the SCFAs acetate and butyrate (64).

Overall, the scientific evidence clearly supports the role of whole grain/high-fiber RTECs for promoting gut microbial abundance and species diversity. Whole wheat and wheat bran-rich RTECs and other fiber-rich foods are associated with increases in fiber-fermenting microbiota, which in turn improve overall microbiota composition (e.g., species abundance and diversity), and fermentation metabolites such as plasma and fecal SCFAs. The bacterial production of certain metabolites such as SCFAs (primarily acetate, propionate, and butyrate) can have major metabolic implications for the host as they provide energy to different tissues and are associated with anti-inflammatory and glucoregulatory effects (22). More specifically, wheat fiber-rich foods have been shown to promote *Bifidobacterium* and *Lactobacillus* levels. These prebiotic effects have been seen with wheat fiber doses as low as 6 g/day and are most prominent among low-fiber consumers (22).

The research also shows that the benefits of wheat fiber consumption on gut bacterial diversity tend to show up in a relatively short period of time (i.e., days to weeks), while the long-term effects are less clear as few studies have lasted for more than 12 weeks. Therefore, more long-term studies (≥12 weeks) are needed to better understand the effects of various dietary fiber sources on the gut microbiota and their fermentation metabolites over time. Additionally, more research is needed to better understand how different food processing techniques and other prebiotic components (e.g., ferulic acid) impact the prebiotic properties of RTECs, as well as how changes in gut microbial abundance/diversity and fermentation metabolites translate into human health outcomes. In the last decade, there have been many significant advancements in the understanding of the impacts of fiber-rich foods and isolated fibers on the gut microbiome, but a limitation of this work is that most of this research has been conducted only in healthy adults and also does not account for the major variation in gut microbial composition and responses between individuals. In sum, the research on whole-grain/high-fiber foods and diets indicate many potential benefits on gut microbial composition, but much more robust and standardized research is still needed across different types of RTECs, fiber types, and human populations.

## 4 Appetite control and weight management

The majority of U.S. adults are classified as overweight (73.6%) or obese (41.9%) based on their BMI (65, 66). Given that obesity increases the risk of several serious health consequences, including hypertension, hypercholesterolemia, CVD, and T2D (67), it is imperative to better understand how dietary patterns can support healthy body weight. Even modest weight loss, as low as 5% of body weight, can significantly improve health outcomes (68). In observational studies, the regular consumption of RTECs has been consistently associated with reduced risk for obesity, although the exact mechanisms are unclear (23, 69). Dietary fiber from different sources may work through different mechanisms to impact energy intake and body weight. For example, soluble fiber from fruits, oats and psyllium have been shown to impact satiety by delaying gastric emptying, while wheat fiber, which is primarily a source of insoluble fiber, has been shown to trigger the release of gut hormones that help regulate food intake (69). Notably, the research on satiety mechanisms and weight control do not always agree, likely because satiety research is mostly focused on heavily controlled acute studies of single meals, whereas weight control research involves a much longer timeframe. Given the different types of studies conducted for satiety and body weight research, these topics are reviewed separately below to better understand if and how cereal fiber dependent satiety mechanisms may translate to body weight outcomes (Tables 4, 5).

**Table 4:** Satiety signals and food/energy intake – systematic reviews and/or meta-analyses.

Author, year	Articles included	Population	Food/fiber type	Measures	Findings
Williams 2014 (23)	8 intervention studies	Healthy adults	Ready-to-eat cereals	Satiety and hunger	Most, but not all studies showed high-fiber ready-to-eat cereals associated with improved satiety and reduced hunger.
Priebe et al. 2016 (24)	7 intervention studies	Healthy adults	Ready-to-eat cereals	Satiety and food intake	Mixed effects of ready-to-eat cereal on satiety and food intake.
Mah et al. 2023 (71)	136 intervention studies	Healthy adults	Extracted and isolated fibers; including AXOS	Appetite ratings and energy intake	Mixed effects of fiber on hunger, and satiety. Viscous fibers and oat bran/fiber associated with improved satiety.

**Table 5:** Body weight regulation – systematic reviews and/or meta-analyses.

Author, year	Articles included	Population	Food/fiber type	Measures	Findings
Williams 2014 (23)	24 intervention studies and 31 observational studies	Healthy adults	Ready-to-eat cereals	BMI, weight loss, risk for overweight and/or obesity.	Observational studies consistently showed ready-to-eat cereals associated with beneficial effects on body weight. Intervention studies were less consistent.
Priebe et al. 2016 (24)	3 intervention studies	Healthy adults	Ready-to-eat cereals	Body weight	No consistent effects of ready-to-eat cereal on body weight regulation reported.
Sanders et al. 2023 (89)	14 intervention studies and 14 observational studies	Adults	Ready-to-eat cereals	Body weight	Observational studies showed ready-to-eat cereals associated with beneficial effects on body weight. Intervention studies showed ready-to-eat cereals were associated with weight loss when consumed as a meal or snack replacement within a hypocaloric diet.
Sanders et al. 2023 (90)	5 intervention studies and 20 observational studies	Children and adolescents	Ready-to-eat cereals	Body weight	Observational studies showed ready-to-eat cereals associated with beneficial effects on body weight. Intervention studies showed mixed results.

### 4.1 Satiety signals and food/energy intake

Growing evidence indicates that dietary fiber intake plays a critical role in appetite regulation and food/energy intake. It has been proposed that dietary fiber’s primary mechanisms of action on appetite suppression work through adding low-calorie bulk to the diet (thereby displacing energy-dense foods in a dietary pattern with foods that are less energy-dense yet similarly satiating), reducing the rate of gastric emptying, and slowing nutrient uptake – which all may impact the release of satiety signals such as glucagon-like peptide 1 (GLP-1), peptide YY (PYY), and cholecystokinin (CCK) (69–71). Although there are many reasonable hypotheses as to how higher fiber intake can impact satiety signaling and reduce overall food/energy intake, clinical trials focused on the effects of whole grain/high-fiber RTECs on appetite regulation provide inconsistent results (69, 72–75).

Given the inconsistent research findings on RTECs, satiety, and food/energy intake, a more nuanced approach may be necessary to elucidate the mechanistic roles of different dietary fiber sources on gut-brain physiology. For example, several studies have shown that the fermentation of prebiotic fibers can alter appetite control in both healthy and obese adults, suggesting that the effects of fiber intake on satiety may include several different signaling cascades and extend throughout the entire GI tract to impact brain signaling (76–79). Moreover, recent research and public health interest strongly suggest that the satiety hormone GLP-1, which is also an incretin hormone involved in insulin secretion and gastric emptying may be a key player mediating the role of high-fiber foods on appetite control and food/energy intake (80). One mechanism of action may be through the effects of fermentable dietary fibers on the gut microbiota, which can increase colonic fermentation metabolites like SCFAs in circulation that can then promote GLP-1 secretion (81). Further research is needed to understand how different food sources, types, and combinations of fibers impact endogenous GLP-1 secretion and their role in promoting satiety and aiding in weight management.

In 2014, a systematic review of RTEC consumption and general health found that most RTEC and satiety research up to 2014 were short-term studies focused on oat-based cereals, hypocaloric diets, and/or refined grain cereals that tracked the effects of RTEC consumption on subsequent meal intake (23). While most of the RCTs included in the 2014 systematic review showed a beneficial effect on satiety and hunger from RTEC consumption, the paper concluded that the results were inconsistent as there was also considerable evidence showing that daily food/energy intake was not reduced by RTEC consumption. Likewise, a 2016 systematic review of RTEC consumption and general health (24), which had a greater focus on whole grain/high-fiber RTEC consumption than the aforementioned 2014 publication, also reported mixed findings on satiety based on the findings from RCTs. The studies from the 2016 systematic review that did show beneficial effects of wheat-based RTECs (73, 82–84) were usually seen with RTEC doses of around 20–30 g/day of insoluble wheat fiber, resulting in approximately 100 to 200 kcal reductions in subsequent energy intake at later meals when compared to low-fiber RTECs containing ≤3 g fiber/serving.

Since 2016, there has been limited research focused on the impacts of high-fiber/whole grain RTECs on satiety and food/energy intake; however, a few reviews have focused on cereal fiber consumption and satiety. A 2022 comprehensive review which investigated the effects of soluble fiber intake from cereal grains on gut hormones found that results from acute trials on GLP-1 were inconclusive, although there was some evidence among healthy populations for increased GLP-1 and PPY levels in long-term studies (80). Similar findings were not seen among long-term studies with overweight populations, or populations with T2D. Notably, beta-glucan, which is found in significantly higher levels in oats than in wheat was linked with increased levels of CCK, which may in turn suppress the hunger hormone ghrelin. The authors of the 2022 review on cereal fiber and satiety hormones concluded that soluble fibers such as those found in resistant starch and beta-glucan are likely the most promising types of fibers for impacting gut hormones due to their prebiotic effects which may impact SCFA production (80). A 2023 systematic review of 136 RCTs (107 acute studies, 29 habitual intake studies) which focused on dietary fiber type, satiety, and energy intake also concluded that most types of fiber are not linked to significant effects on satiety or energy intake (71). The main exception to this finding that was reported was that the habitual intake of resistant starch consistently showed beneficial effects on satiety scores. Additionally, the 2023 systematic review concluded that viscous soluble fibers, such as those from oats and psyllium showed greater satiety benefits than non-viscous fibers, but that the fermentability of fibers had little to no effect on satiety scores (71). This finding about the limited effects of the fermentability (i.e., prebiotic effects) of dietary fibers on satiety seems to contradict other reports, indicating that more research is clearly needed on the impacts of dietary fibers on satiety and food/energy intake.

Several other RCTs provide further evidence of the potential effects of AXOS on GLP-1 response. A 2015 RCT showed that in overweight women, consumption of a high-fiber RTEC enriched with 15 g of AXOS resulted in significant increases in postprandial GLP-1 and PYY after 2 hours, relative to a low-fiber isocaloric control RTEC (85). However, no differences in subsequent meal energy intake were reported, indicating that while a 15 g dose of AXOS may improve satiety signaling, it does not necessarily contribute to lower energy intake throughout the day among overweight women. A 2016 RCT of healthy young adults showed that a single meal of bread containing AXOS-rich wheat bran extract and high-amylose resistant starch led to increases in glucose tolerance, but no changes in PYY or GLP-1 levels, when compared to a white wheat flour bread control (60). And in contrast to both of the above studies, in 2020, a 12-week long RCT on healthy subjects with slow GI transit time that provided 15 g/day of AXOS (in 5 g doses at breakfast, lunch, and dinner), showed an overall decrease in postprandial GLP-1 levels compared to a maltodextrin placebo (47).

Overall, the clinical research on RTECs and cereal fiber consumption on satiety has been mixed and difficult to compare due to the heterogeneity of study designs. In particular, the data on different types of dietary fiber sources and resulting impacts on GLP-1 and other satiety hormones and food/energy intake are sparse and often contradictory. While the impact of foods on satiety hormones can generate hypotheses on that food’s ability to promote satiety, they remain biomarker studies that require verification from long-term studies on food/energy intake. Notably, very few studies on RTEC or cereal fiber intake and satiety or food/energy intake have lasted for more than 3 months. One of the few studies to report on the long-term effects of RTEC intake and satiety was a 2010 RCT that focused on hyperinsulinemic subjects (74). Researchers compared the effects of consuming a high-fiber, wheat-based RTEC (24 g fiber/serving) vs. a low-fiber control (0.5 g fiber/serving) daily on GLP-1 and SCFAs over the course of an entire year. The researchers reported that sustained SCFA and GLP-1 levels were evident only for the last months of the study, which suggests that both repetitive and long-term insoluble cereal fiber intake may be necessary to see consistent satiety and glucoregulatory benefits. Overall, the research on high-fiber RTECs and other fiber-rich foods shows that different types of cereal fibers are likely to act on different satiety mechanisms and different time courses, with the consumption of viscous soluble fibers like oat fiber and psyllium seeming to have the greatest short-term satiety effects. The satiety benefits of wheat fiber are less clear or consistent, potentially because they interact over a longer time course.

### 4.2 Body weight regulation

Due to the complexity of factors involved in body weight regulation, it is difficult to narrow down the impacts of a single nutrient (e.g., fiber), food source (e.g., RTECs), or food group (e.g., cereal grains) on body weight control or body composition. For instance, studies have shown that fiber-rich foods and supplements may upregulate satiety hormones to a greater degree than low-fiber/refined grain foods or other placebo controls, buy these changes do not always translate into higher rating of satiety or reduced food/energy intake (69). Therefore, further investigation regarding the consumption of RTECs and their constituent fibers on body weight regulation is warranted to better understand the role they may play on weight management and obesity risk.

The role of RTEC consumption in obesity prevention has been the subject of several systematic reviews and meta-analyses in the last few decades. Much of this information was summarized in a 2014 systematic review on breakfast cereal and general health (23) that included the findings from three other systematic reviews (86–88) and approximately 30 additional studies focused on weight management. The observational research from this 2014 publication, which primarily came from cross-sectional studies, suggests that individuals who regularly eat breakfast cereals, regardless of their fiber content, tend to have lower BMI and are less likely to be overweight when compared to individuals who do not regularly eat breakfast cereals. On the contrary, the clinical finding were far less consistent and showed mixed results, likely owing to their short duration, narrow focus on next-meal intake, different types of RTECs studied, and different control groups and background diets (23). For example, several of the intervention studies included in the review reported that RTEC consumption could aid weight loss when used as a snack or meal replacement, but most of the clinical intervention studies did not show weight loss benefits when RTECs were added to breakfast. Overall, the 2014 systematic review, which included information from over 50 studies (6 cohort, 30 cross-sectional, 22 RCTs) on breakfast cereal consumption and weight management concluded that the evidence suggests a greater potential protective effect of higher fiber RTECs relative to low-fiber options, but there was still insufficient evidence at that time point to make recommendations on the best types of breakfast cereals for improving body weight control (23).

In 2016, a systematic review focused on RTECs and general health was published which included seven RCTs that specifically examined the impacts of low-fiber vs. whole grain/high fiber RTECs on body weight (24). The studies included in this 2016 publication mainly focused on wheat bran-enriched RTECs, AXOS-enriched RTECs, and beta-glucan enriched RTECs. When prospective cohort studies were reviewed for body weight data, researchers found that frequent RTEC consumption, regardless of their total fiber content, was associated with modest reductions in weight gain and/or lower risk of being obese in both children and adults. Overall, the 2016 systematic review concluded that regular consumption of both low-fiber and high-fiber RTECs may support weight control among normal weight populations, but whole grain and fiber-rich options such as those which have been enriched with bran may better help with long-term body weight regulation by helping to reduce energy consumption throughout the day (24).

In 2023, two highly-focused systematic reviews on the topic of RTECs and body weight were produced by Sanders et al.; one which focused on adults (89) and one which focused on children and adolescents (90). The systematic review on adults included 28 studies (3 prospective cohorts, 11 cross-sectional, and 14 RCTs) and came to similar conclusions as the two previously mentioned systematic reviews from nearly a decade prior (23, 24). Specifically, the results from observational studies showed that regular consumption of RTECs (≥ 4 servings/week), including both low-fiber and high-fiber options, were generally associated with lower weight gain over time, lower BMI scores, and lower prevalence of obesity (89). On the other hand, RCTs showed more mixed and nuanced results often indicating neutral or mildly beneficial effects of RTEC consumption. For example, the RCTs on adults reviewed by Sanders et al., covered many different types of RTECs (e.g., high-fiber, low-fiber, oat-based, wheat-based, corn-based), but only showed potential weight control benefits when RTECs were consumed in a specific manner, i.e., in the form of meal replacements and as part of an energy-restricted diet (89). Sanders et al. also reported that many of the trials did not last long enough to see significant or sustained weight loss or body composition changes and suggested that longer intervention periods (≥6 months) are needed. The authors also recommended including a greater focus on whole grain/high-fiber RTECs in intervention trials as these foods possess unique properties that may better support body weight and composition improvements relative to refined or lower-fiber RTECs (89).

The 2023 systematic review by Sanders et al. on RTEC consumption and body weight among children and adolescents, included 25 studies (2 prospective cohorts, 18 cross-sectional, and 5 RCTs) (90). The results and conclusions from this systematic review were similar to the findings for adults (89). The majority of observational studies showed moderate and beneficial associations between RTEC consumption and body weight, BMI, and/or body composition outcomes, regardless of whether the study participants were consuming presweetened, low-fiber, or high-fiber options. Overall, the body of evidence across the lifespan indicates that regular RTEC consumption, whether from low-fiber or high-fiber RTECs, was consistently associated with favorable or neutral effects on body weight management, although the mechanisms of action are still unclear.

Individual studies of RTEC consumption and obesity risk that were not included in the above systematic reviews offer some additional insights on the effects of dietary fiber type on body weight regulation. A 2017 longitudinal study that followed middle-aged women from Australia over a 12-year period found that higher consumption of both oat-based and wheat-bran (i.e., All-Bran) cereal was associated with significantly lower obesity risk than lower consumption levels (91). In this study, the highest oat-based cereal consumption was associated with 19% lower odds of becoming obese, and highest All-Bran consumption was associated with a 28% lower risk of becoming obese, relative to the lowest consumers.

Studies which have focused exclusively on psyllium-enriched foods or supplementation offer further insights into the role of viscous soluble fiber on body weight control. For instance, a 2023 systematic review and meta-analysis of six RCTs, reported significant benefits of psyllium intake on reductions in body weight and waist circumference among overweight and obese subjects (92). The study reported that these benefits were apparent at mean doses of ~11 g/day of psyllium, over ~5 months of study, and when the doses were given directly before meals. To date, most of the research on psyllium intake and body weight regulation has focused on psyllium supplements and requires greater attention to fully understand the contributions of psyllium-enrich foods like RTECs on body weight control and obesity risk.

In sum, there are several limitations to the body of research focused on RTECs and body weight regulation which make it challenging to differentiate between the effects of different types of RTECs on obesity risk. For instance, many of the studies reviewed on RTECs did not control for physical activity levels or history of dieting behaviors, which are critical confounding factors involved in weight change. There are also contrasting findings between observational and experimental studies, with most observational studies reporting benefits of RTEC consumption on body weight, while RCTs did not consistently do so. Importantly, most of the RCTs that reported on RTEC intake and obesity risk were relatively short-term studies and may not have lasted long enough to account for the small but additive effects that higher-fiber intake may have on reducing appetite and lowering energy intake over a period of several months to years.

Therefore, overall conclusions on RTEC, cereal fiber, wheat bran consumption, and psyllium intake on weight control and obesity risk are difficult to make, especially given that several studies showed that regular consumption of low-fiber and high-fiber RTECs are both associated with lower obesity risk. However, it can be concluded that RTECs and other whole wheat foods that provide adequate amounts of insoluble fiber (i.e., ≥20 g/day), and/or the addition of soluble fiber from sources such as psyllium (i.e., ≥10 g/day), may be effective strategies to help promote satiety and reduce energy intake among both normal weight and overweight populations.

## 5 Cardiometabolic health

Cardiometabolic diseases such as CVD and T2D, which are both largely diet-dependent and obesity-related chronic conditions, are two of the top causes of mortality and morbidity in the U.S. (93). Burgeoning research shows that increasing the consumption of fiber-rich foods could help stem the progression of these largely preventable diet-related chronic diseases, with noticeably greater cardioprotective benefits coming from cereal fiber intake relative to fiber coming from other food groups (94–97). However, many questions remain regarding the most effective cereal fiber type(s), dosing, and timing for the prevention and/or management of different cardiometabolic disease states such as CVD and T2D (Tables 6, 7).

**Table 6:** Cardiovascular disease – systematic reviews and/or meta-analyses.

Author, year	Articles included	Population	Food/fiber type	Measures	Findings
Williams 2014 (23)	75 intervention studies and 7 observational studies	Healthy adults	Ready-to-eat cereals	Blood lipids, blood pressure, coronary heart disease, heart failure, ischemic heart disease, myocardial infarction	Intervention studies showed ready-to-eat cereals that are rich in soluble fiber are associated with improved blood lipid profiles. Observational studies showed whole-grain and bran-based ready-to-eat cereals associated with protective effects on CVD mortality outcomes.
Priebe et al. 2016 (24)	10 intervention studies and 3 observational studies	Healthy adults	Ready-to-eat cereals	Blood lipids, blood pressure, heart failure,	Intervention studies showed ready-to-eat cereals that are rich in soluble fiber are associated with improved blood lipid profiles. Observational studies showed whole-grain/high fiber ready-to-eat cereals associated with protective effects against hypertension and heart failure.
Hajishafiee et al. 2016 (102)	14 observational studies	Adults	Cereal fiber	CVD mortality	Cereal fiber was associated with lower risk for CVD mortality.
Barrett et al. 2019 (52)	11 observational studies	Adults	Cereal fiber and bran	Blood lipids, blood pressure, stroke, coronary heart disease	Cereal fiber and bran were both associated with similar protective effects against CVD.
Zhu et al. 2023 (105)	22 intervention studies	Adults with cardiometabolic disease	Cereal bran	Blood lipids, blood pressure	Cereal fiber associated with improved blood pressure and blood lipid profiles, especially in populations with obesity, lipid disease, and/or hypertension.
Yao et al. 2023 (104)	20 observational studies	Adults	Cereal, fruit, and vegetable fiber	CVD mortality	Cereal fiber associated with lower risk for CVD mortality to a greater degree than fruit or vegetable fiber.
Ramezani et al. 2024 (19)	64 observational studies	Adults	Cereal, fruit, vegetable, nut and seed fiber	CVD mortality	Dietary fiber from all sources associated with reduced risk for CVD mortality.

**Table 7:** Blood glucose management and type 2 diabetes – systematic reviews and/or meta-analyses.

Author, year	Articles included	Population	Food/fiber type	Measures	Findings
Williams 2014 (23)	11 intervention studies and 9 observational studies	Healthy adults and adults with T2D	Ready-to-eat cereals	Blood glucose and insulin, T2D risk	Whole-grain/high-fiber ready-to-eat cereals associated with a reduced risk of developing T2D. Limited evidence from intervention studies linking whole-grain/high-fiber ready-to-eat cereals with improved hyperglycemia in adults with T2D.
Priebe et al. 2016 (24)	6 intervention studies and 2 observational studies	Adults	Ready-to-eat cereals	Postprandial glucose and insulin, T2D risk	Whole-grain/high-fiber ready-to-eat cereals associated with beneficial effects on glucoregulatory factors and lower risk of developing T2D.
Wang et al. 2019 (122)	8 observational studies	Adults	Whole grains and cereal fibers	T2D risk	Whole grain and cereal fiber both associated with reduced risk for developing T2D.
Neuenschwander et al. 2019 (123)	Umbrella review of 53 meta-analyses	Adults	153 different dietary factors, including cereal fiber intake	T2D risk	Cereal fiber shown to be one of the major dietary factors associated with reduced risk for developing T2D.
Hardy et al. 2020 (124)	40 intervention studies	Adults	Carbohydrates, including total fiber and cereal fiber	T2D risk	Cereal fiber associated with reduced risk for developing T2D.

### 5.1 Cardiovascular disease

Approximately 2,500 people in the U.S. die from CVD every day, making it the deadliest disease in the U.S. (98). Major modifiable and diet-dependent risk factors for the development of CVD include hypercholesterolemia, hypertension, obesity, and T2D. Low dietary fiber intake is associated with increases in all of these risk factors and is likely one of the main nutritional factors contributing to CVD development (99). Among U.S. adults, the consumption of cereal fiber has been associated with lower incidence of CVD risk markers relative to other dietary sources of fiber (95, 100). Although the beneficial effects of whole grain cereal intake on cardiovascular health are thought to be provided by their bran fraction and its high content of fiber, there are also several other micronutrients and bioactive compounds in whole grain cereals (most of which are also concentrated in the bran fraction) that may contribute to cardiovascular benefits. In particular, the magnesium and vitamin E content of whole grains have been linked to reductions in oxidative stress and hypertension (24). The bioactive compounds in whole grains such as phytoestrogens, ferulic acid, and other phytochemicals with antioxidant and/or anti-inflammatory properties have also been linked to improved cardiovascular health, and it is proposed that the combined effects of consuming these compounds together with their cereal fiber intact may lead to greater cardiovascular benefits than consuming the cereal fiber alone in isolated form (52, 101). In other words, the health benefits provided by a whole grain (or whole bran) food matrix are likely greater than the sum of their individual parts. Given these potential food matrix effects, whole grain and bran-enriched RTECs are hypothesized to provide greater cardioprotective effects than low-fiber/refined grain RTECs.

In 2014, a systematic review reported that regular consumption of RTECs was not associated with improved total or LDL cholesterol levels unless the RTEC was rich in soluble fiber from sources like oat, barley, and psyllium (23). No consistent effects were reported for the consumption of any high-fiber RTECs on HDL cholesterol levels. However, multiple cohort and case–control studies included in the review reported beneficial effects from higher whole-grain and bran-based RTEC consumption in general on CVD mortality outcomes (i.e., heart failure, ischemic heart disease, myocardial infarction). Likewise, a 2016 systematic review, which included 13 studies on CVD, also found that the type of whole grain cereal fiber matters for CVD markers and outcomes; with RCTs indicating that only soluble fiber-containing RTECs improved lipid levels, and prospective studies indicating that higher intake of all types of whole grain/high fiber RTECs offer protective benefits against hypertension and heart failure (24).

Since the publication of the above-mentioned 2014 and 2016 systematic reviews, additional systematic reviews and/or meta-analyses have been published which have evaluated the impacts of consuming cereal fiber and bran on CVD risk. Most of these reviews only reported on total cereal fiber or bran intake and did not separate out their findings based on insoluble or soluble fiber sources, making it difficult to tease out the unique protective properties of different types of RTECs. For example, a 2016 systematic review and meta-analysis of 14 prospective cohort studies reported that higher consumption of cereal fiber was associated with an 18% risk reduction in CVD mortality, but did not speculate as to whether these effects were more prominent among oat-based or wheat-based foods (102). Likewise, a 2019 systematic review of 11 observational studies found that higher whole grain, cereal fiber, and bran consumption from different sources were all similarly linked with lower risk of negative CVD-related health outcomes (52). These benefits included a reduction in stroke incidence, reductions in serum lipids, improved blood pressure, and reduced progression of coronary atherosclerosis in certain populations. This systematic review also noted that several studies which focused on the impacts of higher bran intake (either from oat or wheat), showed the strongest associations with CVD and coronary heart disease-related benefits, indicating that the components in the bran layer are predominantly responsible for the cardioprotective benefits that whole grains have to offer (52, 103).

A 2023 systematic review and meta-analysis of 20 prospective cohort studies which investigated the role of different dietary fiber types (e.g., vegetable, fruit, cereal, soluble and insoluble) on cardiovascular mortality, found significant beneficial associations for all fiber types (104). For cereal fiber intake, it was reported that the highest daily consumers had a 13% lower risk for CVD mortality relative to the lowest consumers. Dose–response analysis of nine studies on cereal fiber consumption and CVD mortality also found a 16% lower risk per 10 g/day increment of cereal fiber consumption. Dose–response analysis of six studies that focused on insoluble fiber intake and CVD mortality showed a 19% lower risk per 10 g/day increment of insoluble fiber consumed. Furthermore, a dose–response analysis of five studies on soluble fiber intake and CVD mortality revealed a 38% reduction per 10 g/day increment of soluble fiber consumed. A 2023 systematic review and meta-analysis which included 22 RCTs found that higher cereal bran consumption from many different sources including wheat, barley, oat, and rice could help reduce systolic blood pressure, diastolic blood pressure, total cholesterol, and LDL cholesterol – but had no apparent effects on HDL cholesterol or triglyceride levels (105). A 2024 systematic review and meta-analysis of 64 prospective cohort studies also showed that higher intakes of a variety of different fiber sources could reduce CVD mortality, and in contrast to several other reports indicated that the consumption of insoluble fiber tended to be more cardioprotective than soluble fiber consumption (19).

Studies which have investigated the role of psyllium (i.e., viscous soluble fiber) intake on cardiovascular disease offer further insights into the potential benefits of different dietary fiber types on blood pressure and cholesterol levels. In the last decade, multiple systematic reviews and meta-analyses of RCTs reported significant reductions (~2 mmHg) in systolic blood pressure (SBP) with a median dose of 8 g/day of psyllium (106–109). One study reported that each 5 g/day increment in soluble fiber intake, up to 20 g/day, was associated with reductions in SBP (106), while another indicated that of the five types of soluble fiber tested, only psyllium was associated with reductions in blood pressure (109). These studies generally indicated that psyllium’s blood-pressure lowering effects were more substantial among subjects with higher baseline blood pressure levels. Dating back over three decades, systematic reviews and/or meta-analyses of RCTs on psyllium intake and cholesterol levels provide evidence that psyllium intake has total and LDL-cholesterol lowering effects, which are most prominent among individuals with high baseline cholesterol levels (110–113). No effects are generally seen on HDL-cholesterol levels. These studies also suggest that doses in the range of 10 to 15 g/day appear optimal, leading to average reductions of 7–12% in LDL-cholesterol levels (111, 113, 114). Overall, these findings suggest that habitual psyllium intake can support the management of hypertension and hypercholesterolemia, thereby helping to reduce cardiovascular disease risk.

In the last decade, there have been several systematic reviews and meta-analyses that have focused on the effects of RTECs, cereal fiber, bran consumption, and/or psyllium intake, on cardiovascular markers and outcomes. These publications cover more than half a century of research and collectively show that higher dietary fiber intake from many different sources can improve CVD markers and mortality outcomes. Deeper analysis of dietary fiber types generally reveal that the specific types of fibers play different roles in modulating CVD risk; with higher cereal fiber intake generally showing greater cardioprotective benefits than fiber from other food groups; higher bran consumption generally linked with lower risk for CVD mortality than lower bran consumption; and higher psyllium and viscous soluble fiber consumption generally associated with the significant reductions in systolic blood pressure and lipid markers such as total and LDL cholesterol. Overall, there has been very little research published in the last decade specific to whole grain/high-fiber RTEC consumption and CVD markers or outcomes. While the evidence is clear that higher cereal fiber and psyllium fiber consumption is beneficial for cardiovascular health, substantially more research is needed to better understand the role of high-fiber RTEC consumption on CVD outcomes, especially for RTECs that have been enriched with different types of bran, or psyllium, or a combination thereof.

### 5.2 Blood glucose management and type 2 diabetes

It is estimated that prediabetes impacts more than one of every three adults in the U.S., and that over 80% of adults with prediabetes are unaware of their condition (115). If left unchecked, many individuals with prediabetes will progress to having T2D, which is a leading cause of morbidity and mortality in the U.S. (116). For several decades, dietary fiber intake has been linked to improved glucose control in subjects both with and without T2D (117). Among populations without T2D, those with the highest fiber intake generally show a 15 to 19% reduced risk of developing T2D relative to the lowest fiber consumers. Among individuals with T2D, achieving a fiber intake of ≥35 g/day is associated with a 10 to 48% reduced risk of premature mortality (21). When comparing the impacts of different fiber-rich food groups on T2D risk, cereal fiber appears to be more protective effects than fiber from fruits or vegetables (118–121). Several mechanisms have been suggested for how cereal fiber consumption can support glucoregulatory function and reduce the risk for, and progression of T2D. These mechanisms include lowering the glycemic index of carbohydrate rich foods, slowing the absorption of ingested lipids, improving gut microbial profiles, and improving insulin sensitivity by reducing inflammatory markers that are linked with insulin resistance (21, 117). While a large body of research provides evidence for the benefits of total fiber and cereal fiber on blood glucose regulation, much less is known regarding the effects of consuming whole-grain/fiber-rich RTECs on blood glucose regulation, T2D risk, or T2D management.

More than a decade ago, a 2014 systematic review on RTECs and general health reported that there was enough evidence to conclude that higher intake of whole-grain/high-fiber RTECs were associated with a lower risk of developing T2D, however, at that time the evidence was limited linking high-fiber RTECs with improved management of hyperglycemia in individuals with T2D (23). Similarly, a 2016 systematic review reported that evidence from prospective cohort studies and RCTs suggested that habitual whole grain/high-fiber RTEC consumption had beneficial effects on T2D risk (24). Their analysis of prospective studies showed that higher consumption (between 2 and 7 servings/week) of whole grain/high-fiber RTECs was associated with 24 to 40% lower risk for developing T2D when compared to the lowest consumption levels. Their analysis of RCTs found beneficial impacts on glucoregulatory markers for the intervention groups consuming whole-grain/high-fiber RTECs (24). In this systematic review, impressive glucoregulatory benefits were seen among healthy individuals when additional soluble fiber doses around 5 g/day were added to RTECs (24). Therefore, the addition of soluble fiber from oat bran or psyllium to wheat-based RTECs that are already a rich source of insoluble fiber was suggested as a promising strategy for improving glucoregulatory function above and beyond the benefits of provided by intact whole wheat RTEC consumption alone.

A 2019 meta-analysis of eight observational studies (seven cohort, one case–control) that focused on the consumption of higher amounts of whole grains and/or cereal fibers and the relative risk of developing T2D, found a 32% lowered risk for developing T2D with higher whole grain or dietary fiber intake (122). Three of the studies in this meta-analysis focused on whole grains, three focused on cereal fibers, and two on both whole grains and cereal fiber. Overall, the studies that focused on whole grain consumption showed a stronger correlation with reduced risk for T2D than the cereal fiber studies, but the authors indicated that the relative risk reduction among whole grain and cereal fiber studies were very similar because whole grains and cereal fibers both contain large quantities of similar dietary compounds that may help control plasma glucose levels. Taken together, the findings from this meta-analysis of 435,000 subjects suggest that individuals who regularly consume the highest amounts of whole grains and/or cereal fibers in their daily diets have a significantly lower risk for developing T2D, compared to individuals with the lowest consumption levels.

A 2019 umbrella review which examined over 50 prospective studies that focused on the role of diet in T2D incidence, found that of the 153 dietary factors reported (e.g., diet quality, food groups, macronutrients, micronutrients, beverages), some of the strongest and highest-quality evidence for T2D risk reduction was for higher cereal fiber intake (123). The analysis showed a 25% T2D reduction per increment of 10 g cereal fiber/day. A 2020 dose–response meta-analysis of 40 prospective cohort studies also reported strong preventative effects on T2D for every 5 g increase in cereal fiber intake (124). This analysis showed that while total fiber intake was associated with an 8% lower risk for developing T2D, higher cereal fiber intake was associated with a 17% lower risk against developing T2D. These protective effects were even more prominent for cereal fiber intake among females in the study, showing a 33% lower risk per 5 g increment of cereal fiber consumed.

More recently, a 2024 publication took a novel approach to the question of cereal grains and T2D risk, and used UK Biobank data to explore the effects of different cereal grain intake on the risk of T2D (125). This method allowed the researchers to investigate the effects of consuming individual breakfast cereals (e.g., bran cereal, oat cereal, muesli) on T2D risk, rather than combining all breakfast cereals together into a single group. The analysis showed that for subjects without T2D, those who consumed ≥6 bowls/week of bran-based cereal had a 28% lower risk for developing T2D than those who consumed no cereals at all. Similar protective effects were seen for oat-based cereal consumption. Among subjects with T2D but without CVD or chronic kidney disease (CKD), ≥6 bowls/week of bran cereal intake was associated with similar protective effects against developing future CVD or CKD (which are common comorbidities associated with T2D), suggesting that the daily consumption of high-fiber bran cereal may help reduce the risk of developing T2D, as well as reduce the risk of T2D progression into additional cardiometabolic complications.

In addition to the research on cereal fibers, the research on psyllium and T2D risk markers also provide promising results for improving metabolic health. A 2015 meta-analysis of 35 RCTs on psyllium fiber supplementation showed small but significant reductions in fasting glucose levels and hemoglobin A1C (HbA1c) percentages for individuals with T2D (126). These reductions were seen when 7 to 15 g/day of psyllium supplements were provided in divided doses prior to one or more daily meals (e.g., a 5 g dose before breakfast and dinner). Additional systematic reviews and meta-analyses on psyllium offer further evidence that viscous soluble fiber supplementation may provide multiple benefits for populations with T2D. A 2019 systematic reviews and meta-analysis of 28 RCTs focused on the effects of viscous soluble fiber supplements on glycemic control in subjects with T2D, reported that a dose of ~13 g/day of viscous soluble fiber was able to significantly reduce HbA1C levels, fasting blood glucose and HOMA-IR compared to the control group (127). Likewise, a 2021 systematic review and meta-analysis of 29 RCTs which focused on soluble fiber supplements and glycemic control in adults with T2D, reported that only ~8 g/day of soluble fiber supplementation was needed to see improvements in glycemic control (128). A 2023 systematic review and meta-analysis of 17 RCTs which focused on viscous soluble fiber intake and glucose and lipid metabolism among subjects with T2D, also reported that viscous soluble fiber supplementation of around 8 g/day could improve several glucoregulatory markers as well as total and LDL cholesterol levels, with greater improvements seen after 6 weeks of supplementation (129). Furthermore, a 2024 systematic review and meta-analysis of 19 RCTs which focused on psyllium fiber supplementation and glucoregulatory markers in both healthy and unhealthy adults (e.g., adults with T2D or other cardiometabolic disorders) showed that psyllium supplementation significantly decreased fasting blood glucose, HbA1c, and homeostatic model of insulin resistance (HOMA IR) levels, with higher dosage and duration of supplementation being key mediators of glucoregulatory benefits (130). For instance, psyllium supplementation interventions for longer than 50 days, with dosing ≥10 g/day, showed the greatest benefits on reducing fasting blood sugar levels, while dosing ≥10 g/day showed the greatest benefits for lowering HbA1c. In this analysis, psyllium supplementation was not shown to impact insulin levels in any meaningful way.

In the last decade, there have been several systematic reviews and/or meta-analyses that have focused on cereal fiber or supplemental psyllium intake on glucoregulatory function, T2D risk, and/or T2D progression. Notably, few to none of these systematic or meta-analytic reports have included studies on RTECs. Therefore, it is still unclear as to how whole grain/high-fiber and bran-enriched RTECs may benefit these populations. However, the clinical and observational evidence now suggests that there are unique benefits for the regular consumption of different sources of fiber (e.g., whole foods, enriched foods, supplements) and different types of fibers (e.g., insoluble, viscous soluble) for improving blood glucose control. The research on largely insoluble cereal fiber sources (e.g., whole wheat foods, wheat bran-enriched foods) indicate protective actions, and researchers have proposed that the protective effects of cereal fibers on T2D risk may stem from their influence on gut microbial composition and the generation of SCFAs, as well as through related anti-inflammatory and immune boosting properties (97). The research on viscous soluble fibers suggests primary actions through forming a gel matrix that slows down gastric emptying (129).

Overall, the body of research on dietary fiber and blood glucose management shows that increasing dietary fiber consumption from different sources, especially whole cereal grains, to meet recommendations (~35 g/day of total dietary fiber), as well as getting ≥8 g/day of viscous soluble fiber from sources like psyllium may help support glucoregulatory function. The research also indicates that these effects are more likely to benefit individuals with prediabetes or T2D than those with normal blood sugar levels (21, 131). However, translating this information into RTEC consumption recommendations remains difficult since long-term and high-quality data are lacking. Further studies using RTECs which contain different combinations of soluble and insoluble fibers, as well as longer-term RCTs (>12 weeks), are both necessary to better understand the causal relationships impacting glucose management (117, 131). Also of importance is that weight loss can significantly benefit glucoregulatory function and T2D risk. Therefore, research on RTECs and glucose regulation that also includes an energy restriction component, may present an optimal dietary strategy for improving glucose control, T2D risk, and/or T2D management (21).

## 6 Discussion

Overall, a growing body of scientific evidence indicates that obtaining adequate cereal fiber from the diet is associated with improvements in gut function, gut microbial composition, body weight control (in the context of an energy restricted diet), and reductions in the pathophysiology of cardiometabolic disease (22, 38, 52, 54, 131). RTEC consumption, which is one of the primary sources of fiber in North American diets, has also been associated with significant health benefits (23, 24, 89). While several questions remain regarding the most effective whole grain/high-fiber RTECs (e.g., wheat-based, oat-based, bran-enriched, AXOS-enriched, psyllium-enriched) to consume for improving different health outcomes, it is becoming increasingly apparent that the consumer’s health and habits also play a critical role in how RTECs impacts health; with those consumers who initially have low-fiber intake, longer intestinal transit time, lower gut microbial diversity, and/or worse cardiometabolic health markers generally experiencing greater health benefits from whole grain/high-fiber RTEC consumption.

The research insights on RTECs and cereal fibers are also limited by the fact that most studies do not consider the totality of health-promoting nutrients and bioactive compounds in them, sometimes referred to as “co-passengers” (132). Many of these “co-passenger” compounds have been shown to have potent health benefits on their own that can impact health independently of fiber. Additionally, RTECs are commonly consumed with milk, which in itself is a complex food that comes in many varieties (e.g., whole milk vs. reduced fat milk; lactose-containing vs. lactose-free; animal milk vs. plant-based alternatives), each of which may have unique effects on physiology and health (90, 104). Also of importance is that the multitude of studies included in this review present a massive heterogeneity of study designs, which limits the quality of comparisons and conclusions across studies. A large portion of these studies depend on self-reported RTEC or fiber intake data and use methods like food frequency questionnaires or 24-h recalls that are prone to error. Health outcome measures also varied considerably between studies limiting the value of their comparisons.

Furthermore, a growing body of research indicates that a cereal’s food matrix and the way it has been processed may impact its health properties, and this is rarely addressed in the literature when comparing different RTECs. For example, after consuming wheat bran, the bran particle size remains intact and minimally digested in the upper GI tract. It is only once wheat bran reaches the large intestine that certain components, such as the lesser branched forms of arabinoxylans in AXOS start to be fermented by specific gut bacteria and release their phenolic compounds (32). Therefore, the initial bran particle size and texture may be important factors impacting their physiological effects throughout the GI tract (133). Indeed, research has shown that certain types of processing, such as milling, which reduces and smooths out the surface area of wheat bran, may release the phenolic acids from AXOS prior to colonic digestion and also reduce the hydrating properties of bran, which can negatively impact total stool water weight (33). On the other hand, smaller wheat bran particles have been associated with enhanced gut function and improved intestinal peristalsis in animal studies (134). Taken together, this research indicates that larger and coarser (i.e., minimally processed) wheat bran particles may have different health benefits than smaller and finer particles. Therefore, food processing techniques that alter bran’s particle size and structure may impact bran’s functional properties and effects on gut function in multiple ways and deserve further research (32, 133).

Overall, RTECs vary in their ingredients, processing, and nutritional value. All grain foods, including whole-grain/high-fiber RTECs, undergo processing and therefore, are considered to be processed foods. However, multiple systematic reviews have shown that the habitual consumption of RTECs, even those that are not whole-grain or high-fiber are associated with beneficial nutrition and health outcomes (23, 24). Furthermore, a recent systematic review of RTEC consumption on non-communicable disease indicated that there was overall “Grade A” level scientific evidence available showing that the consumption of ultraprocessed cereals and breads were actually associated with improved dietary profiles and reduced risk for cardiometabolic disease (135). Clearly, there is not a direct link between food processing and health when it comes to RTEC consumption, but rather a more nuanced approach is necessary to understand how the overall nutritional and bioactive contributions and food matrices in which they are delivered impact health (136, 137).

Despite all of the variables and limitations of the body of research on RTECs, cereal fibers and health, the evidence examined in this review did allow for several generalizations on the overall effects of consuming whole grain/high-fiber RTECs, cereal fiber, wheat bran, and psyllium on various health outcomes. Furthermore, the safety dossier on cereal fiber intake is extensive, showing a long history of safe use. Indeed, none of the systematic reviews reported long-term negative effects from increasing fiber-rich RTECs, cereal fibers, wheat bran, or their sub fractions of AXOS. To improve on the body of research on whole grain/high-fiber RTECs and health, significantly more long-term RCTs are needed among populations with varying health attributes (e.g., healthy, overweight, hypertensive, insulin resistant), specifically as to the effects of RTECs and cereal fiber-rich foods on the gut microbiome. This type of research may help with improving future health outcomes and health claims by teasing out which physiological effects are due to different fiber sources’ functional properties in the upper GI tract and which effects are associated with colonic fermentation in the lower GI tract. A better understanding of these types of differences could support improved health messaging for different populations and help to close the fiber gap among all populations.

## 7 Conclusion

The roles that whole grains and dietary fiber can play in improving gut health and reducing chronic disease risk have been a focus of nutrition research for decades (138, 139). Research continues to show that soluble and insoluble sources as well as fermentable and non-fermentable sources of fiber provide unique and complementary benefits to human health, and that a diversity of sources of whole grains and cereal fibers are associated with favorable effects on bowel function, microbiome composition, body weight control (in the context of energy-restricted diet), and cardiometabolic health. The findings from this review, which primarily focused on research published in the last 10 years, provide several insights that support the role of whole grain/high-fiber RTECs (which include both intact and enriched sources of fiber) in dietary guidance, health claims, public health initiatives, and dietetic practice.

For decades, it has been hypothesized that the combination of fiber, nutrients, and bioactive compounds in a whole grain food matrix produce additive or synergistic effects that are responsible for the health benefits of whole grains (5, 101, 140). Therefore, whole grains have traditionally been promoted as the ideal source of cereal fiber in the diet. Yet, research also shows significant health benefits when just the bran portion of cereal grains are consumed (54, 141, 142) or when just psyllium is consumed (108, 114, 130). These findings indicate that intact whole grain foods may not always be the most effective or efficient options for increasing dietary fiber intake and improving fiber-related health outcomes. Rather, there is also an important role for bran-enriched foods and psyllium-enriched foods to play in helping people meet their fiber demands and health goals. All in all, there is now substantial scientific evidence available to conclude that adequate cereal fiber consumption from an array of sources can have beneficial effects on health with the most plausible mechanisms of action linked to improvements in gut function and microbial composition. Efforts should therefore continue to encourage increased consumption of cereal fiber-rich foods, such as whole grain/high-fiber RTECs, to help close the fiber gap and promote population health.
